# A graph-theoretic approach for inparalog detection

**DOI:** 10.1186/1471-2105-13-S19-S16

**Published:** 2012-12-19

**Authors:** Olivier Tremblay-Savard, Krister M Swenson

**Affiliations:** 1 Département d'Informatique (DIRO), Université de Montréal, H3C 3J7, Canada; 2 McGill Centre for Bioinformatics, McGill University, H3C 2B4, Canada

## Abstract

Understanding the history of a gene family that evolves through duplication, speciation, and loss is a fundamental problem in comparative genomics. Features such as function, position, and structural similarity between genes are intimately connected to this history; relationships between genes such as orthology (genes related through a speciation event) or paralogy (genes related through a duplication event) are usually correlated with these features. For example, recent work has shown that in human and mouse there is a strong connection between function and inparalogs, the paralogs that were created since the speciation event separating the human and mouse lineages. Methods exist for detecting inparalogs that either use information from only two species, or consider a set of species but rely on clustering methods. In this paper we present a graph-theoretic approach for finding lower bounds on the number of inparalogs for a given set of species; we pose an edge covering problem on the similarity graph and give an efficient 2/3-approximation as well as a faster heuristic. Since the physical position of inparalogs corresponding to recent speciations is not likely to have changed since the duplication, we also use our predictions to estimate the types of duplications that have occurred in some vertebrates and drosophila.

## Introduction

Gene duplication and subsequent modification or loss is a fundamental biological process that is well known to create novel gene function [1]. The first step in most multi-gene studies is to infer the historical relationship of the genes in question; *orthologous *genes are related through a speciation event in the history while *paralogous *genes are related through duplication events. Due to the accelerated rate of divergence of genes after duplication events [2-5], it is generally understood that a pair of paralogous genes are likely to have diverged more than a pair of orthologous genes. Certain recent paralogs, however, may not have had time to diverge significantly. Therefore, paralogs can be further categorized into those that have been created since a particular speciation (*inparalogs*), and those that were created before the speciation (*outparalogs*) [6].

If the speciation in question is a relatively recent speciation then inparalogs represent recent duplications. Thus, they have been used to study properties of duplications under the assumption that the inparalogs have not had time to significantly diverge from the state directly following the duplication [7,8]. Another recent study has shown that for mouse and human, sequence identity for inparalogs is the strongest predictor of gene function (*e.g*. much stronger than orthology) [9].

This motivates the study of large-scale detection of inparalogs. Tree-based inference such as reconciliation is considered to be the most accurate and comprehensive way to infer gene relationships [10-12]. However, large scale application of such methods has historically been limited due to the large amount of computation that must be done to obtain an accurate gene and species tree, from which the reconciliation can be calculated.

Thus, many studies rely on tools based on pairwise similarity measures between genes. Although a daunting number of tools have been developed for orthology detection (due to its relationship to function) [10,13], relatively few have been conceived with inparalog detection specifically in mind. To our knowledge, the only methods that explicitly consider inparalog detection are InParanoid [14], MultiParanoid [15], OrthoMCL [16], and OrthoInspector [17], all of which employ the same basic methodology for inparalog detection: the best similarity between genes in different genomes is evidence for orthology, inparalogs are then inferred to be all pairs of genes within one of the genomes that are more similar than the putative ortholog pair. MultiParanoid has a clustering method built on top of the InParanoid framework to deal with multiple genomes.

In this paper we simultaneously consider the global information given by multiple genes in multiple genomes; this extra information affords us the power to detect less similar pairs of inparalogs, and provides robustness against gene loss. In particular, our approach gives a lower bound on the number of inparalog pairs, based on finding an "orthogonal edge cover" of the colored similarity graph proposed in Zheng et al. [18]. In this graph, each vertex represents a gene and its color represents the genome it belongs to. The edges represent the similarity (*e.g*. sequence, domain, structure, regulatory, isoform, etc.) between the genes. The idea behind our method is to cover the maximum number of genes by orthology relationships. The genes that are left uncovered are considered to have arisen through duplication, and any such gene that is similar enough to a covered gene is considered to be inparalogous to that gene.

The covering step of the method corresponds to finding a so-called maximum orthogonal edge cover of the graph, a problem first posed for finding functional ortholog sets [18]. We propose two algorithms for this optimization problem: one approximation algorithm which covers at least 2/3rds of the number of vertices of a maximum orthogonal edge cover, and a heuristic that is shown to be faster and more efficient on dense graphs.

We apply our method to the genomes of human, chimpanzee, mouse, rat, zebrafish, pufferfish, *Drosophila melanogaster*, and *Drosophila simulans*. We show compelling examples of inparalogs that would not be detected by the other methods (*e.g*. InParanoid). Finally, we show that the distribution of the physical distance between inparalog pairs that we compute is consistent with that of Ezawa et al. [8].

## Inparalogs and multiple species

Given species *A *and *B*, inparalogs are pairs of genes such that one was duplicated from the other since the speciation separating *A *and *B*. The pairwise nature of this definition has led to tools like InParanoid which consider only two genomes at a time. We later motivate the use of many species when inferring inparalogy relationships. In particular, we show that by considering more than two species at once we can 1) be robust to gene loss, and 2) find low similarity inparalogs.

We generalize the definition of inparalogy to consider multiple species with a known phylogeny. For a set of species *S*, any duplication occurring on a branch connected to a leaf gives rise to an inparalog pair for *S*. A *lowest speciation *for *S *is a speciation on the species tree of *S *that has no more recent speciation with regards to the species from *S*.

**Definition 1**. *An inparalog pair for a species set S is a pair of genes (a, b), such that a was duplicated from b after a lowest speciation for S*.

**Figure 1:**
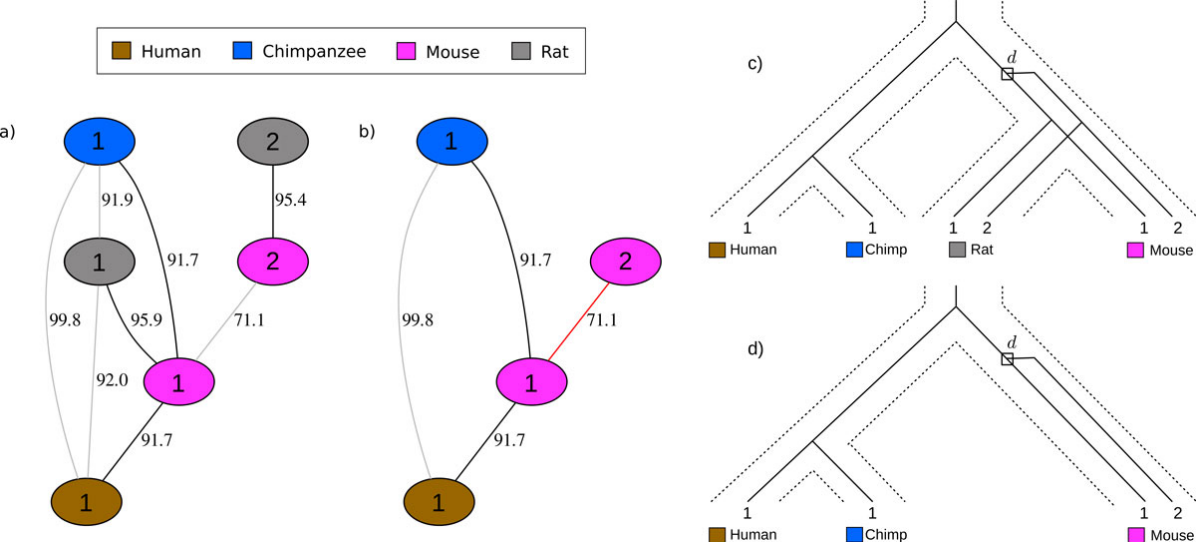
**Inparalogs and multiple species**. (a) is a connected component of the similarity graph (see Section *Experiments on real datasets *for a description of the data) consistent with the gene history depicted in (c). With the complete information from all four species there are no inparalogs inferred, as the duplication *d *is above the speciation between mouse and rat. (b) is the subgraph of (a) with rat removed from consideration. One inparalog is inferred in the mouse with respect to human and chimp (as depicted in (d)).

In the genealogy of Figure 1(c), there are no inparalogs since the duplication d occurred before the speciation of mouse and rat. On the other hand, the duplication *d *in Figure 1(d) does correspond to an inparalog pair since it occurred after the speciation between mouse and human/chimp. Note that Definition 1 is a generalization of the traditional definition.

## Inparalogs and edge covers

InParanoid builds sets of inparalog pairs which it then must merge based on an extensive set of rules. We forgo this complicated merging process by considering the pairwise similarities in a global fashion. Further, our method is robust to gene loss due to the fact that we consider the genes from multiple genomes at once. Consider the graph *G*(*V*, *E*), where *V *has one vertex per gene and *E *has an edge *e = *(*v*, *u*) with weight *w*(*e*) corresponding to the similarity of gene *v *and *u*. The vertices are colored by the genome that they come from. We refer to *G *as the *similarity graph*. Figure 1(a) shows a component of the similarity graph for human, chimp, mouse, and rat, along with the simplest gene history consistent with this information. The similarity graph holds the global information corresponding to the history of all gene families. An important property about the genes in the similarity graph is the following. Call any maximal set of genes that originated from the root or from a duplication event an *ortholog set*. For example, Figure 1(c) shows two ortholog sets: the leaves labeled 2 originate from the duplication d, while the leaves labeled 1 originate at the root of the tree.

**Property 1 **(orthogonality [18]). *Any ortholog set corresponds to a subgraph of G where there exists at most one vertex with a given color*.

**Figure 2:**
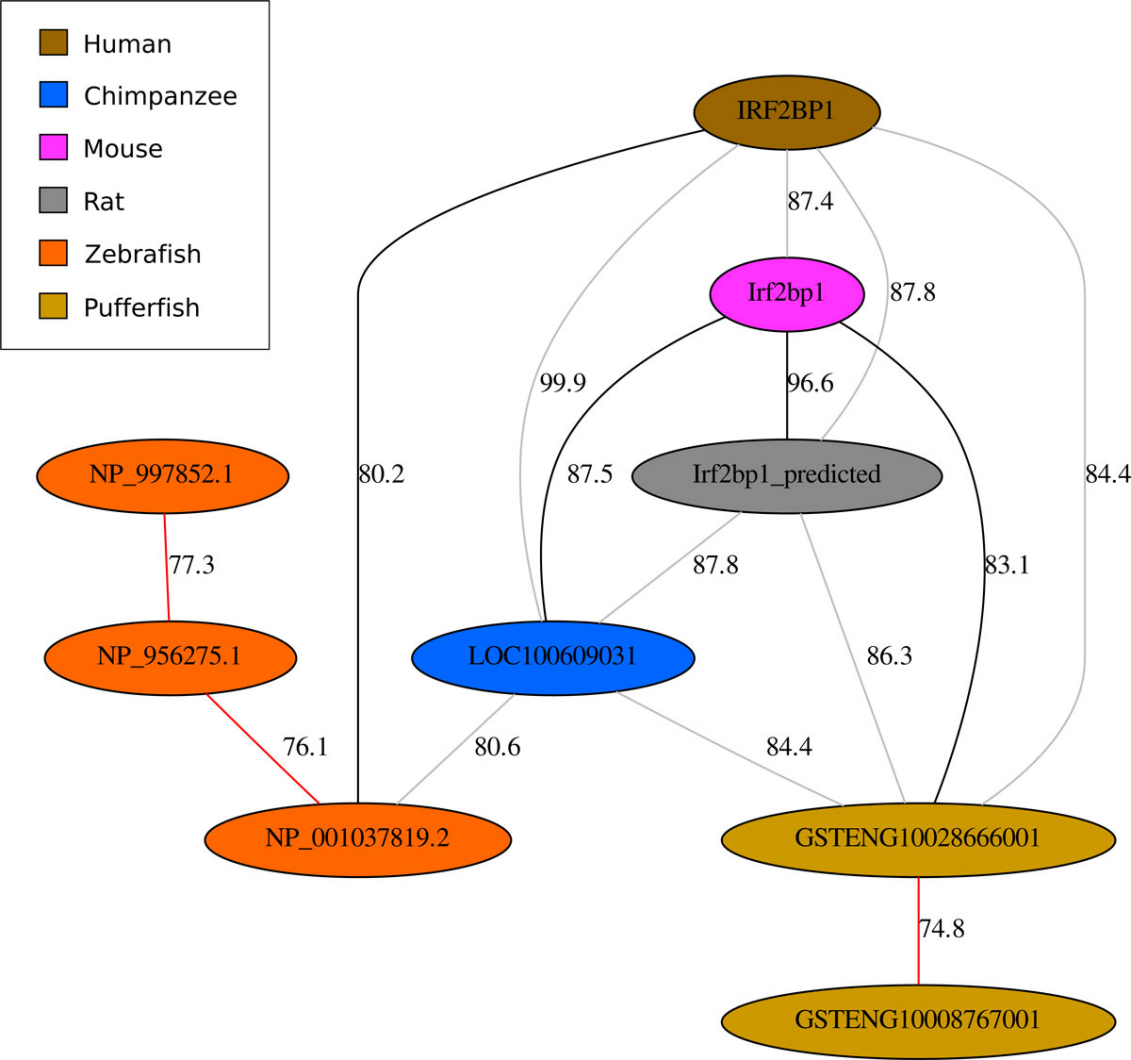
**A connected component of the similarity graph**. See Section *Experiments on real datasets *for a description of the data. Black edges are part of our cover while red edges highlight inferred inparalog relationships. Despite the low similarity between genes NP_956275.1 and NP_001037819.2, a clear signal for their inparalogy is present. The relationship is confirmed by gene annotations. InParanoid does not detect such inparalogy for the speciation between zebrafish and pufferfish.

An *orthogonal subgraph *of *G *is a graph *G' *= (*V*, *E'*) where *E' *⊆ *E *and every connected component is orthogonal. We can consider the edges E´ to be the evidence for orthology sets. In the similarity graphs of Figures 1 and 2, each component induced by only the black edges is orthogonal whereas the graph with all edges is not.

Our method is based on the observation that inparalogs belong to orthology sets of size one, whereas in the absence of losses all other paralogs will be orthologous to at least one other gene. Figure 1(d) depicts a history where there is only a single copy of gene 2 in the mouse which is inparalogous to gene 1 of the mouse, whereas Figure 1(c) has no single copy gene and no inparalogs.

Thus, for a given subgraph of *G *we consider genes that have at least one edge incident to them to be *covered*, since covered vertices represent those genes that are orthologous to at least one other gene. This motivates the following approach:

1. find an orthogonal subgraph of *G *such that the maximum number of vertices are covered, and then

2. mark as inparalogs all uncovered vertices with high similarity to some other gene in the same genome.

Step 1 corresponds to solving the maximum orthogonal edge cover problem. In Section *Maximum orthogonal edge cover *we present an $O\left(\left|V{|}^{\left(1.5\right)}|E\right|\right)$ 2/3-approximation algorithm for this problem, along with a faster and simpler heuristic. Step 2 is implemented in two different ways. The first way calls an uncovered gene inparalogous to the highest weight neighbor that is covered, provided that weight is above some threshold. The second considers the possibility that a chain of duplications originated from a single gene, and is described in the next section.

### Chains of duplications

A chain of multiple duplications, each originating from the previous duplicate copy, will result in multiple uncovered vertices of a single color, as depicted for zebrafish in Figure 2. For this reason, we have an indirect version of step 2 that builds a maximum spanning tree of uncovered vertices under the premise that they are all inparalogs. In the example of Figure 2 the maximum spanning tree happens to be a path of orange vertices.

### Further motivation

The simplest notion of inparalogy requires only a single genome and a measure of similarity between genes: the most closely related genes would then just be the proposed inparalogs. For example, Ezawa et al. [8] used the synonymous distance and a threshold to identify recently created paralogs. InParanoid uses a little more information by including two genomes and taking genes as inparalogs if their similarity is greater than the best orthology assignment for either one. We motivate the need for a global approach with two examples from the data in Section *Experiments on real datasets*, where we applied our algorithm to the whole genomes of two great hominoids, two rodents, two fish, and two flies.

Figure 2 shows an example where a pair of inparalogs for a low scoring pair is detected by our algorithm. In particular, genes NP_956275.1, NP_001037819.2, and NP_997852.1 are marked as inparalogous with respect to the speciation between zebrafish and pufferfish. The genes from human, chimp, mouse, rat, along with NP_001037819.2 from zebrafish, are all annotated as "interferon regulatory factor 2-binding protein 1". Remarkably, NP_956275.1 is annotated as "interferon regulatory factor 2-binding protein 2-A", and the beginning of the gene NP_997852.1 is annotated as "Interferon regulatory factor 2-binding protein zinc finger". The genes for the inparalogy marked in pufferfish have yet to be annotated in the NCBI database. Note that, at 77.0, the similarity between NP_001037819.2 from zebrafish and GSTENG10028666001 from pufferfish is below our threshold of 80 and is not depicted. Since the similarity between NP_001037819.2 and NP_956275.1 is less than this, InParanoid does not mark the two as inparalogs with respect to the zebrafish/pufferfish speciation; the InParanoid7 database labels the two as inparalogs with respect to *Drosophila melanogaster*.

**Figure 3:**
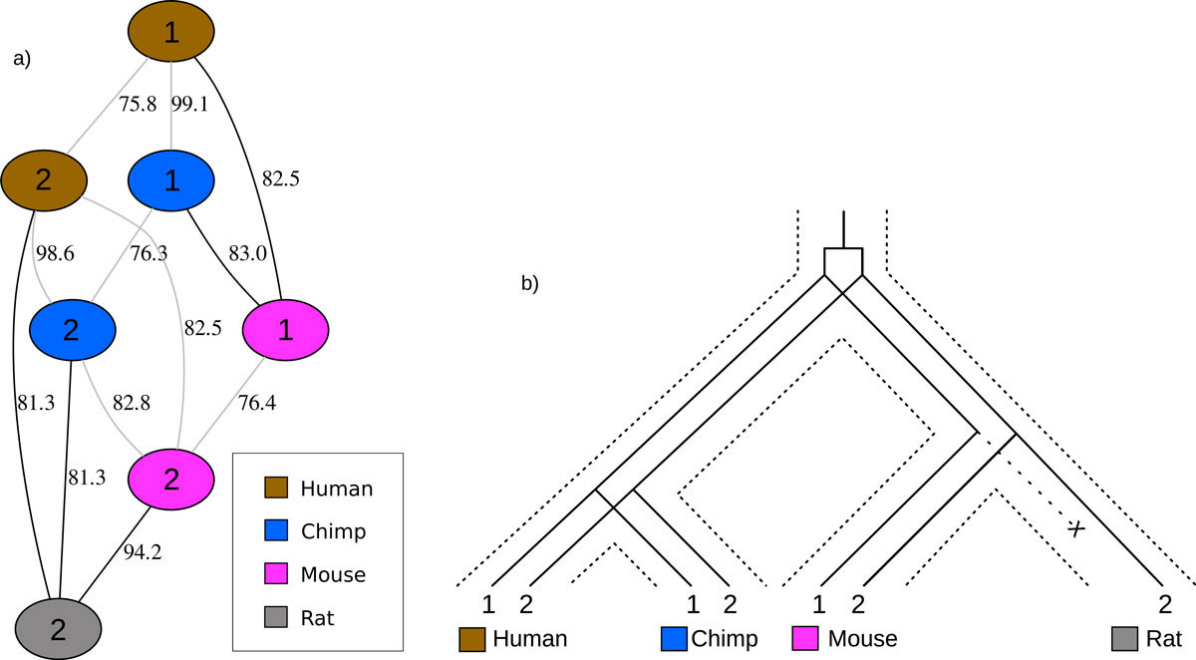
**Losses in the context of multiple genomes**. (a) is a connected component of the similarity graph (see Section *Experiments on real datasets *for a description of the data) consistent with the gene history depicted in (b) representing protocadherin gamma b1 (1) and b2 (2) genes. The protocadherin gamma b1 gene is known to have been lost in rat [34]. It is essential that human or chimp be considered in the analysis with mouse and rat, otherwise a false inparalog relationship would be inferred between the two mouse genes.

A slightly more general, but simpler, approach than that of InParanoid would consider the similarity graph for two genomes; in this case the graph is bipartite. Thus, a maximum matching on the weighted graph covers the maximum number of genes with the maximum amount of global similarity. The uncovered vertices are then candidates to be inparalogs; those that are similar enough to other genes are considered to be inparalogous to those genes. While this method may not suffer from the problem of lower similarity between inparalogs (illustrated in Figure 2), it may suffer from that of Figure 3. In this case we see a simple example where consideration of only two genomes (mouse and rat) will hide the fact that there was a loss of gene 1 in rat, resulting in a false inparalog assignment of genes 1 and 2 in mouse.

### Thresholds

Step 1 of our algorithm calls for a subset of the edges that results in a minimum number of uncovered vertices. Note that this measure does not have anything to do with the number of edges or the weight of the edges that are chosen; the maximum orthogonal partition problem is inherently unweighted. For this reason, our method requires a threshold for interspecies similarity scores; all edges labeled above the threshold will be considered significant. Similarly, to reduce false positives, the intraspecies similarity scores may have a different threshold.

An interspecies threshold that is too high will yield an unweighted graph with components that are very small, and we will lose the power of the multiple genome inference. An interspecies threshold that is too low may yield large components that have too many optimal solutions. While there may be some question as to what threshold is the best, we have yet to do a detailed study on this. Instead, we have chosen conservative thresholds for both measures; all the results reported in this paper have interspecies threshold of 80 and intraspecies threshold of 70.

## Maximum orthogonal edge cover

In this section we describe the algorithms for maximum orthogonal edge cover. Our 2/3-approximation algorithm runs $O\left(\left|V{|}^{\left(1.5\right)}|E\right|\right)$ time. While the heuristic has the same worst-case complexity, we show in Section *Experiments on simulated datasets *that the running times are faster in practice.

Take a set *S *with a color function c:S↦ℕ

**Definition 2**. *An *orthogonal partition *of set S is a partition *U*_i_S_i _*= *S such that for any distinct s*, *r *∈ *S_i_*, *c*(*s*) ≠ *c*(*r*).

Take a graph *G = (V, E) *with a color function c:V↦ℕ. Traditionally, an *edge cover *is a subset *E' *⊆ *E *such that all vertices are present in at least one edge of *E'*; for this we reserve the term *perfect edge cover*. For our purposes, we relax the definition of "edge cover" to be *any *subset of *E*.

**Definition 3**. *An *edge cover *of G is a subset of E*.

Consider the partition of *V *induced by the connected components of an edge cover.

**Definition 4**. *An *orthogonal edge cover *of a graph G is an edge cover where the induced partition on V (by the "is connected to" relation) is orthogonal*.

A maximum orthogonal edge cover of *G *is an orthogonal edge cover which covers the maximum number of vertices, over all possible orthogonal edge covers.

Let *v*(*E*) denote the vertex set for some edge set *E*. The maximum orthogonal edge cover (MAX-OREC) problem can be stated as follows:

**Input**: Undirected graph *G *= (*V*, *E*) and color function c:V↦ℕ.

**Solution**: An orthogonal edge cover *E' *⊆ *E *(*i.e*. for each connected component *C *of *G*' = (*V*, *E'*) we have *c*(*s*) ≠ *c*(*r*) for all distinct *s, r *∈ *C*).

**Measure**: The number of vertices covered (*i.e*. |*v*(*E'*)|)

We present a 2/3-approximation algorithm for MAX-OREC. Our approach is to first compute edges that cover the maximum number of vertices for each color, while ignoring the orthogonality constraint. We then show that the connected components of this edge cover have a particular structure, allowing us to ensure orthogonality without removing too many edges.

### Bipartite matchings

Consider the bipartite graph *B*(*x*) = (*U*, *W*, *F*) where *U *is the subset of *V *with color *x*, *W *= *V \U*, and *F *consists of the edges that span *U *and *W *(*i.e*. *U *= {*v *: *v ∈V*, *c*(*v*) = *x*} and *F = *{(*u*, *w*) : (*u*, *w*) ∈ *E*, *u *∈ *U*, *w *∈ *W*}). The following property on orthogonal edge covers holds.

**Property 2**. *A maximum matching M(x) on B(x) covers the maximum (over any edge cover) number of vertices of color x, without breaking the orthogonality constraint*.

Now take a maximum orthogonal edge cover Q*, and an edge cover consisting of all the edges from all the bipartite matchings R = ∪_i _*M*(*i*). The following is a direct consequence of Property 2.

**Lemma 1**. |*v*(Q*)| ≤ |*v*(*R*)|.

If every connected component of *H *= (*V*, *R*) has an orthogonal edge cover, then |*v*(*R*)| = |*v*(Q*)|. We show in the next section that, while we can not always find an orthogonal edge cover for every component, we can always find an edge cover that includes at least 2/3rds of the vertices in the component.

### Covering bounded degree graphs

Consider the neighborhood of a particular vertex *v *from *H *= (*V*, *R*) with color *x*. Any vertex in the neighborhood of *v *with color *y *is a result of the matching *M*(*x*) or *M*(*y*). Thus, there are at most two vertices of color *y *connected to *v*. We call a graph with this property *2-neighborhood-limited *(2NL). We use the fact that *H *is 2NL to show that we can find an orthogonal edge cover that includes at least 2/3rds of the maximum number of possible vertices.

Call a path in a component *alternating *if vertices in the path alternate between two colors. The *length *of a path is the number of vertices in the path. Then we have the following two lemmas.

**Lemma 2**. *A connected 2NL graph G that contains no odd-length alternating path has a perfect orthogonal edge cover*.

*Proof*. Any even-length alternating path can be covered by taking every other edge on the path (a perfect matching). Consider the graph *G' = (V, E') *where *E' *is *E *without the edges that are removed from perfect matchings on even-length alternating paths. Note that this graph has no alternating paths and that the degree of all vertices is at least one.

Take a minimal edge cover *C' ⊆ E' *that covers all the vertices of *V*. *C*' is composed only of stars (every component has no simple path of length greater than two), otherwise it would not be minimal. Since no edge of *C' *links two vertices corresponding to the same color and there exists no alternating paths in *G'*, the edge cover *C' *must be orthogonal. □

**Lemma 3**. *Each odd-length alternating path can contribute to at most one uncovered vertex in a maximum orthogonal edge cover*.

*Proof*. Every other edge on an odd-length alternating path can be matched, leaving a single vertex uncovered. □

Lemmata 2 and 3 imply the following algorithm for finding an approximate orthogonal edge cover on a 2NL graph where *maximumMatching(H') *returns a maximum matching on *H' *and *minPerfectEdgeCover(H") *returns a minimal perfect edge cover for each component of *H" *that has more than one vertex (*i.e*. a perfect edge cover where removing any edge will result in an uncovered vertex). The correctness of the algorithm follows the same reasoning as the proof of Lemma 2.

**Algorithm 1 **getMAX-2NL-OREC(*H *= (*V*, *R*))

*P *= {the set of edges in alternating paths of *H*}

H' = (v(P), P)

*E" = maximumMatching(H') *∪ *(R\P)*

H" = (V, E")

**return ***minPerfectEdgeCover(H")*

### Bringing things together

Say Algorithm 1 returns an edge cover *Q *for a 2NL graph while an optimal edge cover is Q*. Then the following is a direct consequence of Lemmata 2 and 3.

**Lemma 4**. |*v*(*Q*)| > |*v*(Q*)| - *p where p is the number of odd-length paths in a 2NL graph*.

So counting the number of odd-length paths gives us an idea of how far we could be from the optimal. Since the shortest possible odd-length path has three vertices, and two of them can be covered, we get the desired approximation guarantee.

**Lemma 5. **$\left|v\left(Q\right)|\;>\;\frac{2}{3}|v\left(Q^{*}\right)\right|$

Now, using Section *Bipartite matchings *along with Algorithm 1, we can use Algorithm 2 to approximate the MAX-OREC problem where *M*(*x*) is the maximum bipartite matching between the vertices with color *x *and all the other vertices. Say Algorithm 2 returns an edge cover *O *while an optimal edge cover is O* . Then the following are a direct consequence of Lemmata 1, 4 and 5.

**Theorem 1**. |*v*(*O*)| > |*v*(O*)| **- ***p where p is the number of odd-length paths in a graph*.

**Theorem 2. **$\left|v\left(O\right)|\;>\;\frac{2}{3}|v\left(O^{*}\right)\right|$

**Algorithm 2 **getMAX-OREC(*G*)

*R *← Ø

**for **each color *x *in *G ***do**

*R *← *R *∪ *M*(*x*)

end for

*O *← Ø

**for **each component *C *of *H *= (*V*, *R*) **do**

*O *← *O *∪ getMAX-2NL-OREC(*C*)

end for

**return ***O*

### Running time

The running time of Algorithm 1 is $O\left(\sqrt{\left|V\right|}|E|\right)$ since a minimal perfect edge cover and a maximum matching can be computed in $O\left(\sqrt{\left|V\right|}|E|\right)$ time, while listing alternating paths takes linear time. The running time of Algorithm 2 is therefore $O\left(\left|C|\sqrt{\left|V\right|}|E\right|\right)$, where *C *is the number of colors. In the worst case this bound is $O\left(\left|V{|}^{\left(1.5\right)}|E\right|\right)$ since there are at most O(|*V*|) colors.

### A fast heuristic

We also developed a practical algorithm for MAX-OREC. It is simpler to implement and runs faster in practice and performs better on dense graphs (see Section *Experiments on simulated datasets*). The algorithm does the following:

1. compute *H*, the union over all maximum bipartite matchings for every pair of colors, and then

2. compute *minPerfectEdgeCover*(*H*).

Note that the main difference with the approximation algorithm is that we do not compute the same maximum bipartite matchings.

## Results and discussion

### Experiments on simulated datasets

We implemented the 2/3-approximation algorithm and the heuristic in C++ and we applied them to simulated datasets in order to compare their performance. We generated random graphs using the *G*(*n*, *p*) model introduced by Gilbert [19]. A random graph *G*(*n*, *p*) has *n *nodes and for each *n*(*n *- 1)/2 possible pairs of nodes, an edge is created with probability *p*. The expected number of edges in a *G*(*n*, *p*) graph is $\left(\begin{array}{c} n\\ 2 \end{array}\right)p$. The probability *p *corresponds to the expected percentage of completeness of the random graph.

**Figure 4:**
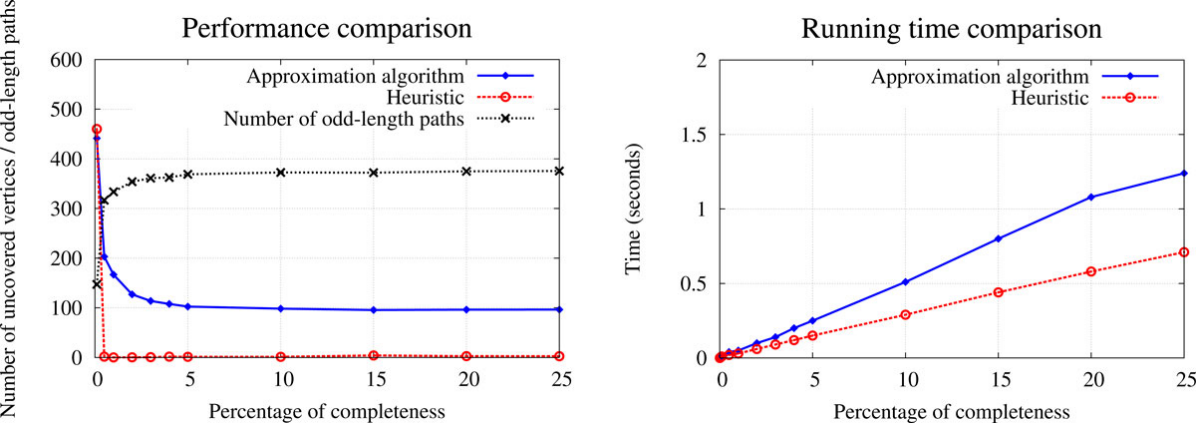
**Comparison of the performance of the 2/3-approximation algorithm and the heuristic**. The results are averaged over 100 random graphs of 2500 vertices (genes) and 5 colors (genomes). Left: Comparison of the number of uncovered vertices. The number of odd-length alternating paths is also shown. Right: Running time comparison.

Figure 4-left shows the number of uncovered vertices (averaged over 100 replicates) given by both algorithms for random graphs of 2500 nodes and a varying expected percentage of completeness. Clearly, the heuristic performs a lot better than the approximation algorithm on dense graphs. It is also faster than the approximation algorithm (Figure 4-right). However, the approximation algorithm covers more vertices than the heuristic on really sparse graphs (under 0.1% of completeness). We also show the average number of odd-length alternating paths that is found by the approximation algorithm. Since the number of uncovered vertices given by the approximation algorithm is always significantly lower than the number of odd-length alternating paths (except for very sparse graphs), it is clear that the approximation algorithm covers more vertices than the worst expected case (*i.e*. 2/3 of the optimal maximum number of covered vertices).

### Experiments on real datasets

In this section, we present an analysis of the inparalog pairs inferred by our approach on the genomes of human, chimpanzee, mouse, rat, zebrafish, pufferfish, *Drosophila melanogaster*, and *Drosophila simulans*. We first describe how we obtained the data and then we show an example of how we can use recent inparalogs to study modes of duplication.

#### Creating the input graph

We used CoGe:SynMap [20] interface to Last (Blast variant) to make all-versus-all pairwise comparisons between the studied species and the self comparisons. SynMap is usually used to find syntenic regions containing a minimum number of genes (block size), but in the context of this experiment, we simply used it with a block size of one to identify all the homologous genes. We discarded similarity edges when one gene in the pair was more than 1.25 times longer than the other.

#### Modes of duplication and recent inparalogs

The most studied duplication mechanisms are whole genome duplication, tandem duplication and retrotransposition. Whole genome duplication has the effect of simultaneously doubling all the chromosomes of a genome. It has been shown that whole genome duplication has occurred at least once [21,22] and maybe twice [23-25] in the vertebrate ancestor. Then, a fish-specific round of genome duplication was reported by studies conducted on teleost fish [26-28] and an additional round was shown to have occurred in the salmonid fish lineage [29]. As the name implies, tandem duplication creates adjacent duplicate gene copies. It is believed that unequal crossing-over during meiosis is the principal mechanism responsible for the creation of tandem duplicates [30]. The third well-studied duplication mechanisms is retrotransposition, which usually produces intronless gene copies that can end up anywhere in the genome. In mammals, LINE-1 retrotranposons are mainly responsible for creating those duplicates [31].

Another mode of duplication that has been receiving more attention in the recent years is the one responsible for the creation of segmental duplications. It has been named duplicative transposition in [32] and drift duplication in [8]. This kind of duplication can create in one step duplicate gene copies (with introns, as opposed to retrotransposition) that are transposed anywhere in the genome, even on different chromosomes. The biological mechanisms behind duplicative transposition are not yet fully understood, but it is believed that Alu repeats could be involved in primates [33].

In order to better understand duplication mechanisms and study the relative rates of the different types of duplications, it is interesting to study recently created gene duplicates. For example, a study on recently emerged paralogs in human, mouse, zebrafish, *Drosophila melanogaster*, *Drosophila pseudoobscura *and *Caenorhabditis elegans *suggested that drift duplication occurs nearly as often as tandem duplication in vertebrates [8].

#### Analysis of the inparalog pairs

**Figure 5:**
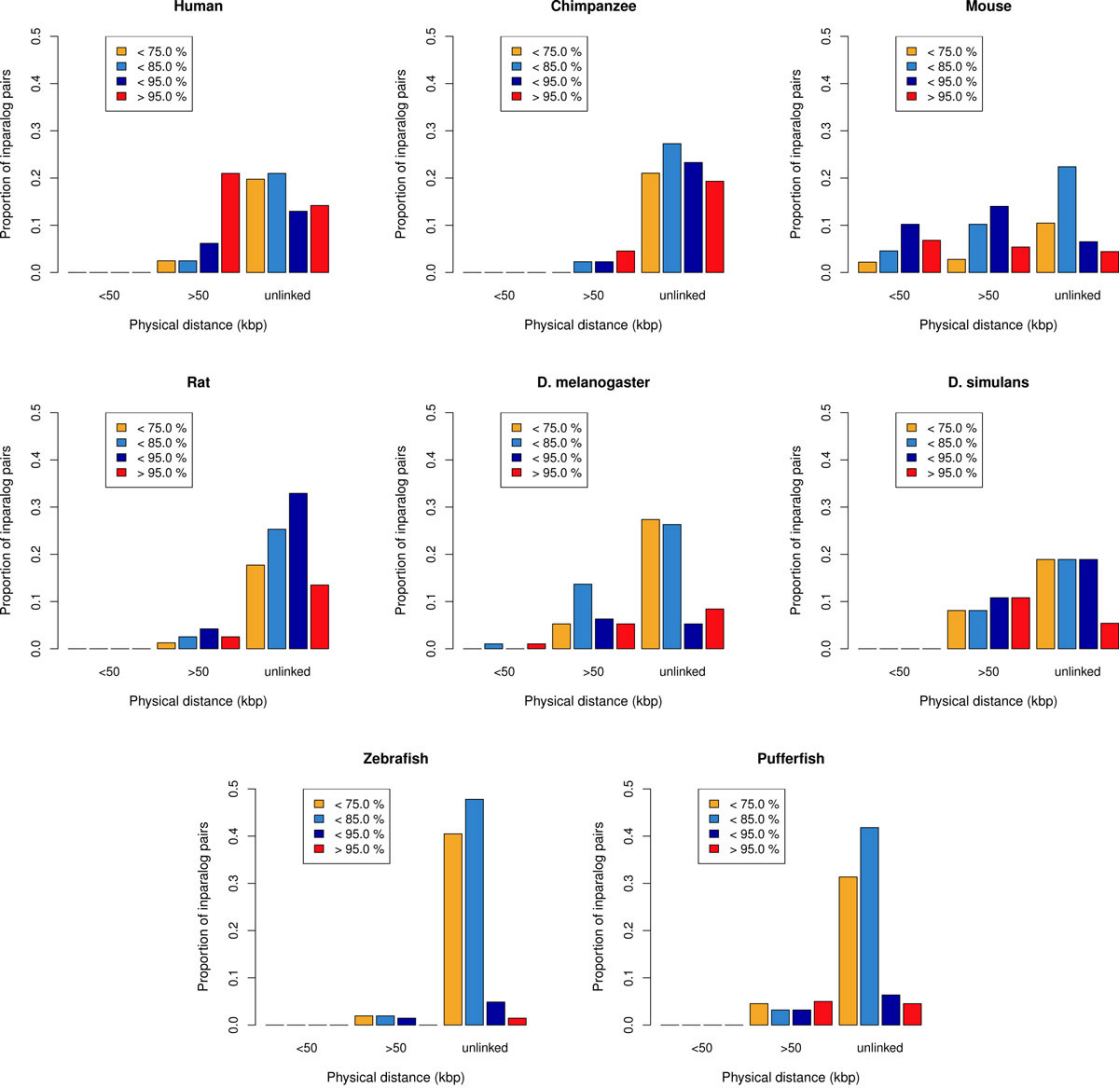
**Proportions of inparalog pairs inferred in the 8 species studied**.

We identified inparalog pairs in the studied genomes and retrieved information on their physical distance and percent similarity. Figure 5 presents, for each species, the distribution of the inparalog pairs for three classes of physical distance and four classes of percent similarity. The three classes of physical distance represent inparalog pairs separated by less than 50 kbp, inparalog pairs separated by more than 50 kbp and unlinked inparalog pairs, *i.e*. inparalog pairs that are located on different chromosomes. The 50 kbp boundary was chosen as an attempt to separate possible tandem duplication events (physical distance of less than 50 kbp) from the other more far-reaching duplication mechanisms (like retrotransposition and duplicative transposition). We used three boundaries to divide the inparalog pairs into four percent similarity classes, in order to verify if there is a correlation between the age of the duplication events and the physical distance.

Only mouse and *D. melanogaster *have inparalog pairs with a physical distance of less than 50 kbp, which suggests that tandem duplication could occur more frequently in those species. Inparalog pairs having a physical distance of more than 50 kbp are rare in chimpanzee, rat, zebrafish and pufferfish, but make for an important fraction of the inparalog pairs of human, mouse and the two drosophilas. The most interesting case is in human, where more than 20% of the >50 kbp inparalog pairs are recent (>95% similarity). This could suggest that a significant number of linked duplicative transpositions or retrotranspositions occurred relatively recently in human, which is consistent with the findings of Ezawa et al. [8].

For all the species, a large fraction of the inparalog pairs are unlinked. This is especially true for zebrafish and pufferfish, where more than 80% of the inparalog pairs are located on different chromosomes. Interestingly, the majority of the unlinked pairs in the fish species have a low percent similarity. We hypothesize that this could be the result of ongoing fractionation after the fish-specific whole genome duplication. Human, chimpanzee and rat all have at least 10% of recent unlinked inparalog pairs (>95% similarity). This could be evidence of recent duplicative transpositions or retrotransposition. Older unlinked inparalog pairs (<95% similarity) do not necessarily correspond to older duplicative transposition events. For example, a scenario involving tandem duplication followed by genomic rearrangement events could have produced the same results.

## Conclusion

We presented a new graph-theoretic approach for the detection of inparalogs. Our method uses a maximum orthogonal edge cover on the similarity graph and then identifies inparalogs in the set of uncovered vertices. We developed a 2/3-approximation algorithm for this problem and a heuristic that was shown to be faster and more efficient on dense graphs. Note that our method is not suitable for finding orthologous gene relationships since our edge covers aggressively leave the minimum number of genes unmatched. Zheng et al. [18] discuss other objective functions on the similarity graph that are more suitable for orthology detection.

We have shown compelling examples of why using the information for multiple species gives more accurate inparalog predictions and how our method allows us to infer inparalogs that would not have been found by other methods like InParanoid. We then presented an example of how we can use recent inparalogs to study modes of duplication. Our analysis of the genomes of human, chimpanzee, mouse, rat, zebrafish, pufferfish, *D. melanogaster *and *D. simulans *suggested that many recent tandem duplications occurred in mouse and that a significant number of linked duplicative transpositions or retrotranspositions occurred relatively recently in human.

We did not show speed comparisons with other existing methods like InParanoid because our method was very fast on real data. The results on the real datasets were obtained in 10 seconds on a typical Linux workstation.

On the methodological side, algorithmic improvements that consider edge weights while finding an edge cover are possible, as well as improved preprocessing of the data. The question remains as to which other measures of similarity our method is most powerful with.

On the evaluation side, we attempted to make large-scale comparisons against inparalogy given by reconciliation (Ensembl gene trees), but we were not able to convert in an automated manner a statistically significant number of gene names from SynMap to Ensembl IDs in order to do so. While computing statistics -- like the number of inparalog pairs shared with a method like InParanoid -- are possible, direct comparison as to which method finds the correct inparalog relationships remains difficult since few independent methods or bench experiments exist for finding such relationships.

## Competing interests

The authors declare that they have no competing interests.
